# A comparison of visual and quantitative methods to identify interstitial lung abnormalities

**DOI:** 10.1186/s12890-015-0124-x

**Published:** 2015-10-29

**Authors:** Corrine R. Kliment, Tetsuro Araki, Tracy J. Doyle, Wei Gao, Josée Dupuis, Jeanne C. Latourelle, Oscar E. Zazueta, Isis E. Fernandez, Mizuki Nishino, Yuka Okajima, James C. Ross, Raúl San José Estépar, Alejandro A. Diaz, David J. Lederer, David A. Schwartz, Edwin K. Silverman, Ivan O. Rosas, George R. Washko, George T. O’Connor, Hiroto Hatabu, Gary M. Hunninghake

**Affiliations:** From the Pulmonary and Critical Care Division, Brigham and Women’s Hospital, Harvard Medical School, Boston, MA USA; Department of Radiology, Brigham and Women’s Hospital, Boston, MA USA; Department of Biostatistics, Boston University School of Public Health, Boston, MA USA; The National Heart, Lung, and Blood Institute’s Framingham Heart Study, Framingham, Massachusetts, Boston, MA USA; Department of Medicine, Boston University, Boston, MA USA; Department of Neurology, Boston University, Boston, MA USA; Center for Pulmonary Functional Imaging, Brigham and Women’s Hospital, Boston, MA USA; Channing Division of Network Medicine, Brigham and Women’s Hospital, Boston, MA USA; Surgical Planning Laboratory, Department of Radiology, Brigham and Women’s Hospital, Boston, MA USA; Department of Pulmonary Diseases, Pontificia Universidad Católica de Chile, Santiago, Chile; Division of Pulmonary and Critical Care, College of Physicians and Surgeons, Columbia University, New York, NY USA; Department of Epidemiology, Mailman School of Public Health, Columbia University, New York, NY USA; Department of Medicine, University of Colorado, Denver, CO USA; Pulmonary Center, Department of Medicine, Boston University School of Medicine, Boston, MA USA

**Keywords:** High attenuation areas, Idiopathic pulmonary fibrosis, Interstitial lung disease, Interstitial lung abnormalities (ILA), MUC5B

## Abstract

**Background:**

Evidence suggests that individuals with interstitial lung abnormalities (ILA) on a chest computed tomogram (CT) may have an increased risk to develop a clinically significant interstitial lung disease (ILD). Although methods used to identify individuals with ILA on chest CT have included both automated quantitative and qualitative visual inspection methods, there has been not direct comparison between these two methods. To investigate this relationship, we created lung density metrics and compared these to visual assessments of ILA.

**Methods:**

To provide a comparison between ILA detection methods based on visual assessment we generated measures of high attenuation areas (HAAs, defined by attenuation values between −600 and −250 Hounsfield Units) in >4500 participants from both the COPDGene and Framingham Heart studies (FHS). Linear and logistic regressions were used for analyses.

**Results:**

Increased measures of HAAs (in ≥10 % of the lung) were significantly associated with ILA defined by visual inspection in both cohorts (*P* < 0.0001); however, the positive predictive values were not very high (19 % in COPDGene and 13 % in the FHS). In COPDGene, the association between HAAs and ILA defined by visual assessment were modified by the percentage of emphysema and body mass index. Although increased HAAs were associated with reductions in total lung capacity in both cohorts, there was no evidence for an association between measurement of HAAs and *MUC5B* promoter genotype in the FHS.

**Conclusion:**

Our findings demonstrate that increased measures of lung density may be helpful in determining the severity of lung volume reduction, but alone, are not strongly predictive of ILA defined by visual assessment. Moreover, HAAs were not associated with *MUC5B* promoter genotype.

## Background

Although idiopathic pulmonary fibrosis (IPF), the most common and severe form of interstitial lung disease (ILD), has historically been unresponsive to pharmacotherapy [1, 2], recent studies have finally demonstrated that two medical therapies [3, 4] can reduce the rate of decline in lung function, particularly when started early in the course of disease [5]. These findings provide an important motivation to identify patients in early stages of disease.

Accumulating evidence suggest that interstitial lung abnormalities (ILA) identified on chest imaging may identify groups of subjects at risk to develop clinically significant ILD in general [6], and IPF in particular [7, 8]. Support for this statement comes evidence that participants with ILA are more likely to have respiratory symptoms [9, 10] and physiologic decrements (e.g. reduced lung volumes [9–11], exercise capacity [12], and diffusion capacity of carbon monoxide [DLCO]) [7, 10] suggestive of, but less severe than, those apparent in patients with IPF [13]. In addition, multiple studies have identified radiologic progression in participants with ILA [7, 8, 14], a usual interstitial pneumonia (UIP) pattern on chest computed tomograms (CTs) in some participants with ILA [7–9, 11, 14], and progression from a non-UIP pattern to a UIP pattern in small numbers of subjects [7, 8]. Finally, the genetic factor most strongly associated with IPF (*MUC5B* promoter genotype) [15] has been similarly associated with ILA in the Framingham Heart Study (FHS) [10].

While these findings suggest the potential importance of identifying ILA, and comparisons between quantitative and visual assessments of clinically established pulmonary fibrosis have been performed [16–18], there has been no published data on the relationship between quantitative and visual assessments for ILA identification. In particular, while all studies to date have utilized chest CTs and most have relied on qualitative visual assessment [7–10, 14], one study utilized a quantitative assessment of lung density to identify ILA [11]. Assessing lung density as a method of identifying ILA is attractive as it could be automated, it could provide a continuous metric of the degree of abnormality, and it would be compatible with methods commonly used to identify and quantify the extent of emphysema in research studies [19]. To compare these two methods, we created lung density metrics and compared these to visual assessments of ILA in two large cohorts [9, 10].

## Methods

### Study design

Protocols for enrollment and phenotyping for both the COPDGene and the Framingham Heart Study (FHS) participants have been described previously [9, 10]. In brief, the COPDGene cohort analyzed in this article includes 2508 non-Hispanic White (*n* = 1867, 74 %), and African-American (*n* = 641, 26 %) smokers (with at least 10 pack years of smoking) between the ages of 45 and 80, who were enrolled into COPDGene at 21 clinical centers from November of 2007 to April of 2010. The FHS cohort included in this article includes 2764 mostly non-Hispanic white adult men and women from the Third Generation and Offspring Cohorts who participated in FHS Multi-Detector Computed Tomography 2 (FHS-MDCT2) Study. The COPDGene study (including this ancillary study) was approved by the institutional review boards (IRBs) of all participating centers and FHS-MDCT2 study (and this ancillary study) was approved by the Boston University and the Brigham and Women’s Hospitals’ IRBs (2010P000996 and 2007P000554 for the FHS and COPDGene, respectively). All included participants in both cohorts provided written informed consent, including consent for the use of their DNA in genetic studies.

### Chest CT acquisition protocols

In COPDGene, the CT acquisition protocol used was as follows: 120kVp, 200mAs, and 0.5 s rotation time for General Electric (GE) LightSpeed-16, GE VCT-64, Siemens Sensation-16, Siemens Sensation-64, Philips 40-slice, and Philips 64-slice scanners. Images were reconstructed using a standard algorithm at 0.625 mm slice thickness and 0.625 mm intervals for GE scanners. Siemens CT images were reconstructed using a B31f algorithm at 0.625 (Sensation-16) or 0.75 mm slice thickness and 0.5 mm intervals. Reconstruction of Philips images was performed by using B algorithm at 0.9 mm slice thickness and 0.45 mm intervals. In the FHS, CT images were acquired with a General Electric Discovery VCT 64-slice PET/CT scanner (GE Healthcare) using a MA determined by subject weight (300 mA for subjects less than 220 lbs, 350mG for subjects equal to or greater than 220 lbs) 120 Kv, and a gantry rotation time of 0.35 s. Raw data was collected using a 210° scan reconstruction algorithm and a detector width of 0.625 mm. Images were reconstructed with a 50 cm field of view (FOV).

### Thoracic chest CT analysis

#### Visual assessment

The methods for the visual assessment of chest CTs in both COPDGene and FHS have been described previously [9, 10]. Chest CTs were evaluated by three readers using a sequential reading method as previously described [20]. ILA were defined as nondependent changes (to exclude the effect of atelectasis as prone images were not available) affecting >5 % of any lung zone including, nondependent ground-glass or reticular abnormalities, diffuse centrilobular nodularity, nonemphysematous cysts, honeycombing, or traction bronchiectasis. CTs with either focal or unilateral ground-glass attenuation, focal or unilateral reticulation, or patchy ground-glass abnormality (<5 % of the lung) were considered indeterminate.

### Quantitative assessment

Image analysis was performed on inspiratory CT scans using Pulmonary Workstatins 2 and PLUS (VIDA Diagnostics, Iowa City, IA) at the Core Imaging Center of the COPDGene study to quantify the percentage of the lung occupied by high attenuation areas (HAAs, defined by attenuation values between −600 and −250 Hounsfield Units [HUs]) [11]. While this method includes lung segmentation of both the parenchyma and central vasculature, these vessels are ultimately excluded in quantitation based on the attenuation values of blood (~40 HUs depending on the hematocrit). Airway Inspector (http://airwayinspector.acil-bwh.org) was used to quantify the total lung capacity and percentage of emphysematous lung (at [−]950, in COPDGene) [19].

### Statistical analysis

In both COPDGene and the FHS, the percentage of HAAs was evaluated as both a continuous and as a binary variable selected on the basis of a prior presentation [11] (e.g. ≥10 % or <10 % and ≥6.4 % or <6.4 % [≥6.4 % corresponds to the ≥ upper 95^th^ percentile] of the lung occupied by HAAs in COPDGene). To provide a complete comparison between methods, when ILA was the primary outcome we assessed controls as those that both included and excluded participants indeterminate for ILA by visual assessment. In COPDGene, covariate adjustment and statistical analyses were performed as described using Statistical Analysis Software version 9.2 (SAS Institute, Cary, NC). In the FHS, all analyses accounted for familial relationship using generalized linear mixed effect models as previously described [21] and were performed using R version 2.9 [22]. All genetic analyses were performed using additive genetic models [10]. Reported *P* values are two-sided and those < 0.05 were considered statistically significant.

## Results

### Population characteristics of ILA by characterization method

Population characteristics of participants with ILA, defined by both visual assessment and a threshold of HAAs for both the COPDGene and FHS, are presented in Table 1. Population characteristics of participants without ILA and indeterminate for ILA defined by visual assessment and the interobserver variability of ILA assessments in both the COPDGene and FHS have been presented previously [9, 10]. The most strikingly consistent differences between the classification methods of ILA are that participants with ILA defined by HAAs tended to have an increased BMI and greater reductions in total lung capacity (TLC) in both the COPDGene and FHS cohorts when compared to participants with ILA classified by visual assessment.

**Table 1:** Baseline characteristics of COPDGene and Framingham Heart Study (FHS) participants with Interstitial Lung Abnormality (ILA) by identification method

Variable^a^	Number (%) or Median (standard deviation) where appropriate			
Demographic parameters	COPDGene		FHS	
	ILA by Visual Assessment	ILA by HAAs^b^	ILA by Visual Assessment	ILA by HAAs^b^
	*n* = 194 (8 %)	*n* = 26 (1 %)	*n* = 177 (7 %)	*n* = 46 (2 %)
Age (years)	64 (10)	62 (8)	70 (12)	65 (13)
Gender (female)	101 (52 %)	14 (54 %)	89 (50 %)	19 (41 %)
Race (African-American)	56 (29 %)	14 (54 %)	-	-
Body Mass Index	28 (7)	33 (4)	28 (5)	32 (5)
Pack years of smoking	44 (27)	42 (26)	26 (20)	18 (15)
Current Smoker	97 (50 %)	18 (69 %)	17 (10 %)	5(11 %)
Spirometric Parameters				
FEV_1_ (% of predicted)^d^	81 % (21)	80 % (32)	98 % (17)	96 % (16)
FVC (% of predicted)^d^	88 % (17)	78 % (23)	101 % (15)	96 % (15)
FEV1/FVC %^d^	71 % (14)	81 % (18)	97 % (9)	100 % (7)
DLCO (% of predicted)^e^	-	-	86 % (14)	86 % (17)
Chest CT parameters				
Total Lung Capacity (TLC)^f^	5.0 (1.4)	3.5 (1.4)	4.6 (1.2)	3.8 (0.9)
TLC % of predicted^f^	95 % (20)	57 % (17)	79 % (17)	64 % (14)

^a^Data missing in the COPDGene study (*n* = 2416) for COPD status and pulmonary function testing (*n* = 1, <1 %) and TLC (*n* = 19, <1 %). Data missing in the FHS (*n* = 2633) for spirometry (*n* = 165, 6 %), diffusion capacity of carbon monoxide (DLCO, *n* = 572, 22 %), and total lung capacity (*n* = 192, 7 %)

^b^HAAs = High Attenuation Areas (defined by attenuation values between −600 and −250 Hounsfield Units [HUs]) [11]

^c^Gold Stage ≥ 2 includes those with an FEV_1_/FVC % ≥ 0.70, FEV_1_ < 80 % of predicted

^d^Post-bronchodilator pulmonary function measurements presented. Predicted values for FEV_1_ and FVC are derived from Crapo et al. [34]

^e^DLCO = Diffusion capacity of carbon monoxide, predicted values are derived from Miller et al. [27]

^f^Quantitative metrics of TLC were performed using Airway Inspector (http://airwayinspector.acil-bwh.org). HU: Hounsfield units. Percent of predicted total lung capacity based on ATS/ERS guidelines [35]

### COPDGene

**Fig. 1:**
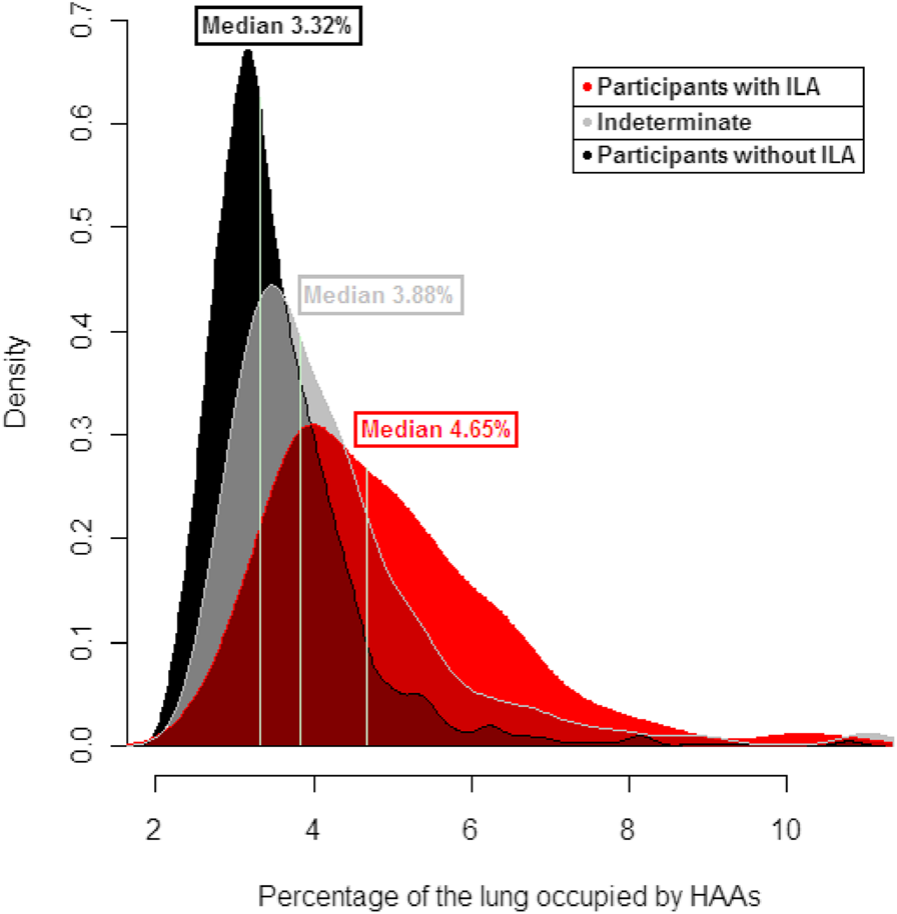
A density plot of the percentage of the lung occupied by High Attenuation Areas (HAAs, chest CT attenuation values between −600 and −250 HU) in participants with ILA (in red, *n* = 163), in participants indeterminate for ILA (in gray, *n* = 757), and in the participants without ILA (in black, *n* = 1173). Despite the differences in numbers between the groups for each category (defined by color) the area under the curve is normalized to a density of 1 which gives a sense of the relative spread of the data between categories. The percentage of lung occupied by various HAA thresholds is listed on the x-axis. The density of subjects at various HAA thresholds is listed on the y-axis

In COPDGene, of the 2416 chest CTs previously assessed for the presence of ILA [9], quantitative assessment of HAA could be measured in 2093 (87 %) participants. HAAs were significantly associated with ILA in analyses that excluded participants indeterminate for ILA status (for each 1 % increase in HAAs a participant had a 2.3-fold increase in their odds to have ILA, 95 % confidence interval [CI] 2.0–2.6, *P* < 0.0001) and in analyses including participants indeterminate for ILA as controls (for each 1 % increase in HAAs a participant had a 1.3-fold increase in their odds to have ILA, 95 % confidence interval [CI] 1.2–1.4, *P* < 0.0001). In addition, increases in HAAs were associated with reductions in total lung capacity (TLC) both in analyses of all participants (after adjusting for covariates including age, sex, race, body mass index [BMI], smoking behavior, the percent of emphysema and COPD status) and in analyses limited to those with ILA (after adjusting for covariates). For all participants, for each 1 % increase in HAAs a participant was predicted to have a 351 ml decrease in TLC (95 % CI 324–378 ml, *P* < 0.0001). For those with ILA, for each 1 % increase in HAAs a participant was predicted to have a 278 ml decrease in TLC (95 % CI 216–340 ml, *P* < 0.0001). However, as noted in Fig. 1, the difference between the median value of the percentage of the lung occupied by HAAs among participants with ILA, compared to those without ILA, was < 1.4 %. Among participants with ILA, the median value of HAAs was 4.6 % ranging between 2.7 and 16.5 %. Among participants without ILA, the median value of HAAs was 3.3 % ranging between 2.2 and 10.8 %.

Next, we assessed the ability of HAA thresholds to predict the presence of ILA by visual assessment in COPDGene. As noted in Table 2, although there was significant evidence for a correlation between ILA defined by visual assessment and those having ≥10 % of the lung occupied by HAAs (*P* = 0.03), the agreement between methods was slight (Kappa = 0.03). The positive predictive value of having ≥10 % HAAs in the prediction of ILA by visual assessment in COPDGene was 19 %. We considered an alternate threshold of lung density (≥6.4 %, corresponding to the upper 95^th^ percent of HAAs in COPDGene), and noted increased evidence for a correlation between ILA defined by visual assessment and lung density methods (*P* < 0.0001). However, the agreement between methods remained modest (Kappa = 0.13). The best performing threshold of HAAs in the prediction of ILA defined by visual assessment (c-statistic 0.76) was obtained with an HAA threshold of ≥ 3.6 % (which corresponds to the 50^th^ percentile of HAAs in COPDGene).

**Table 2:** Comparison of methods for the detection of interstitial lung abnormalities in COPDGene and Framingham Heart Study (FHS) participants

COPDGene	HAAs < 10 %^a^	HAAs ≥ 10 %^a^	HAAs < 95^th^ percentile (HAA < 6.44 %)^a^	HAAs ≥ 95 percentile (HAA ≥ 6.44 %)^a^
No ILA^a^	1909	21	1850	80
ILA	158	5	139	24
	Kappa 0.03, *p* = 0.03		Kappa 0.13, *p* < 0.0001	
Framingham Heart Study	HAAs < 10 %	HAAs ≥ 10 %	HAAs < 95^th^ percentile	HAAs ≥ 95 percentile
No ILA^a^	1987	306	2188	105
ILA	104	46	132	18
	Kappa 0.11, *p* = <0.0001		Kappa 0.08, *p* < 0.0001	
COPDgene Cohort:				
HAAs at 10 %	Sensitivity 3 %	Specificity 99 %	PPV 19 %	NPV 92 %
HAAs at 95^th^%	Sensitivity 15 %	Specificity 96 %	PPV 23 %	NPV 93 %
Framingham Cohort:				
HAAs at 10 %	Sensitivity 31 %	Specificity 87 %	PPV 13 %	NPV 95 %
HAAs at 95^th^%	Sensitivity 12 %	Specificity 95 %	PPV 15 %	NPV 94 %

^a^ *HAAs* high attenuation areas (defined by the percentage of the lung occupied by high attenuation areas between −600 and −250 Hounsfield Units) [11]

^b^Number of subjects grouped as “no ILA” that are classified as indeterminate: COPDgene: HAA <10 % - *n* = 739, HAAs ≥ 10 % - *n* = 18, HAAs < 95 % – *n* = 698, HAAs ≥ 95 % – *n* = 59

^c^Number of subjects grouped as “no ILA” that are classified as indeterminate: Framingham: HAA <10 % - *n* = 805, HAAs ≥ 10 % - *n* = 184, HAAs < 95 % – *n* = 926, HAAs ≥ 95 % – *n* = 63

**Fig. 2:**
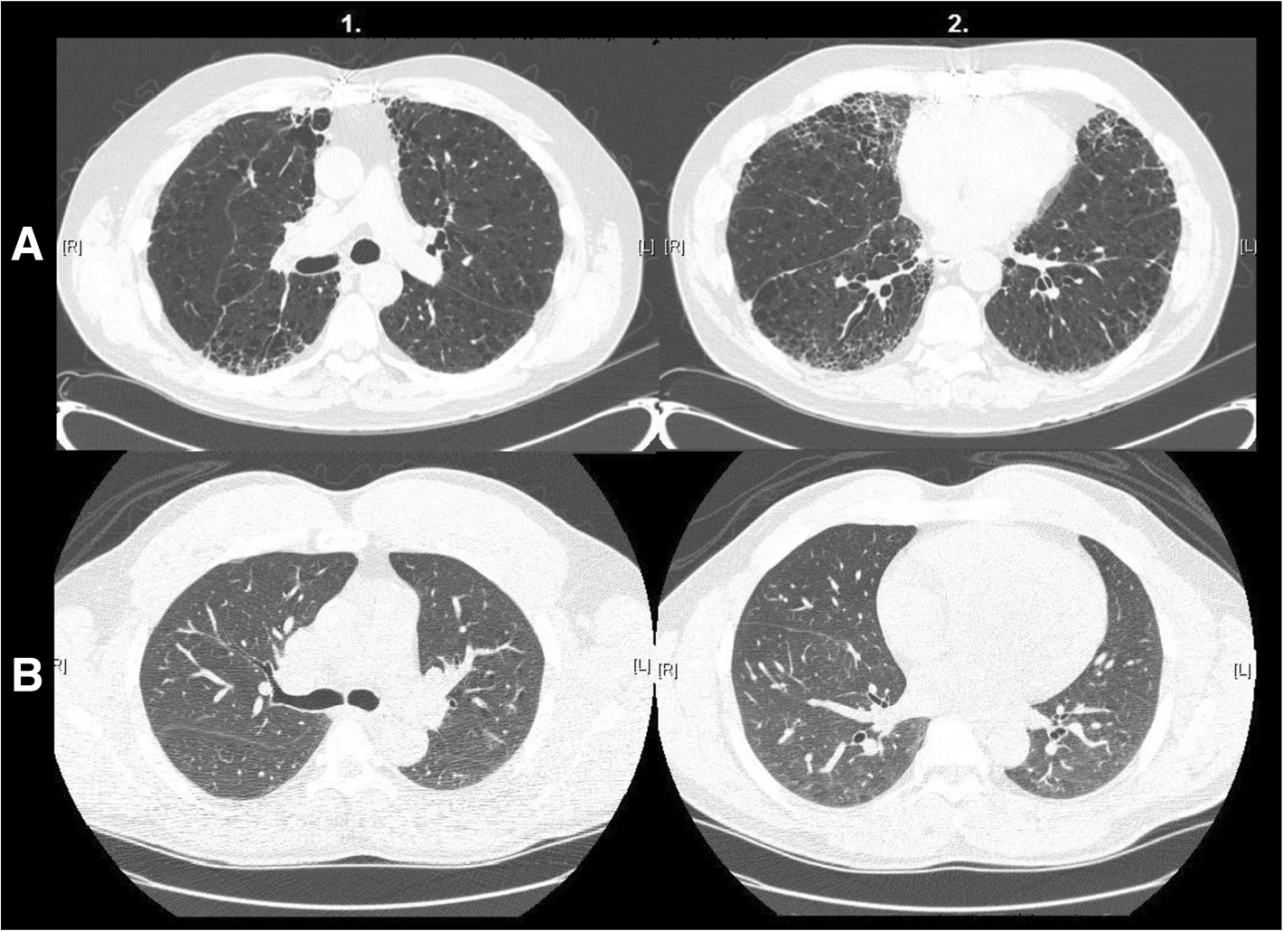
On the vertical axis we present representative examples of (**a**) a subject with interstitial lung abnormalities identified by visual assessment but has less than 10 % of the lung with chest CT attenuation values between −600 and −250 HU (HAA) (7.5 % HAA) and (**b**) a subject having > 10 % of the lung with chest CT attenuation values between −600 and −250 HU (HAA) (10.8 %) but not identified as having interstitial lung abnormalities identified by visual assessment. Each row represents data from a single subject. On the horizontal axis we present axial high resolution chest computed tomographic (HRCT) images (1 [approximately at the level of the carina] and 2 [approximately at the level of the right inferior pulmonary vein])

There is evidence that the association between HAAs and ILA defined by visual assessment was modified by the percentage of emphysema (*P* = <0.0001) and the BMI (*P* = 0.03) of participants. For example, among participants with <5 % emphysema, for each 1 % increase in HAAs a participant had a 1.2-fold increase in the odds of having ILA (95 % CI 1.1–1.3, *P* < 0.0001). Whereas, among participants with ≥5 % emphysema, for each 1 % increase in HAAs a participant had a 3.8-fold increase in the odds of having ILA (95 % CI 2.3–6.0, *P* < 0.0001). Among participants with a BMI ≥30, for each 1 % increase in HAAs a participant had a 1.9-fold increase in the odds of having ILA (95 % CI 1.6–2.3, *P* < 0.0001). Whereas, among participants with a BMI <30, for each 1 % increase in HAAs a participant had a 3.1-fold increase in the odds of having ILA (95 % CI 2.5–4.0, *P* < 0.0001). Figure 2 demonstrates an example of a participant with ILA defined by visual assessment on the background of emphysema but with <10 % HAAs, and an example of a participant without ILA defined by visual assessment and an increased BMI with ≥10 % HAAs. As apparent in Fig. 2 (and noted in the data above), an increase in the percentage of emphysema appears to reduce the sensitivity of HAA thresholds for the detection of ILA by visual assessment, while an increased BMI alone can result in an increase in the percentage of HAAs even when there is minimal visual evidence for ILA on chest CT imaging.

### Framingham Heart Study (FHS)

As a prior study utilizing HAAs in the identification of ILA was performed in a general population sample [11], we additionally analyzed the FHS and compared the association between thresholds of HAAs and ILA defined by visual assessment in 2443 (93 %) of the previously assessed 2633 chest CTs, where this measurement could be obtained.

As noted in Table 2, and comparable to our findings in COPDGene, although there was significant evidence for a correlation between ILA defined by visual assessment and those having ≥10 % of the lung occupied by HAAs (*P* = <0.0001), the agreement between methods was slight (Kappa = 0.11). Comparable to results from COPDGene, increases in HAAs were associated with reductions in TLC both in analyses of all participants (after adjusting for covariates including age, sex, race, BMI, and smoking behavior, for each 1 % increase in HAAs a participant was predicted to have a 233 ml decrease in TLC, 95 % CI 223–243 ml, *P* < 0.0001) and in analyses limited to those with ILA (after adjusting for covariates, for each 1 % increase in HAAs a participant was predicted to have a 214 ml decrease in TLC, 95 % CI 179–249 ml, *P* < 0.0001). However, the positive predictive value of having ≥10 % HAAs in the prediction of ILA by visual assessment in the FHS was 13 %. Similar evidence was noted when we considered an alternate threshold of lung density (≥6.4 %, Kappa 0.08, *P* < 0.0001). The positive predictive value of having ≥6.4 % HAAs in the prediction of ILA by visual assessment in the FHS was 15 %.

Finally, based on a prior association between *MUC5B* promoter genotype (rs3570950) and ILA defined by visual assessment in the FHS [10], we compared the association between *MUC5B* promoter genotype and ILA defined by various thresholds of HAAs and continuous measures of HAAs. After adjusting for covariates including age, sex, body mass index, and smoking behavior, there was no evidence for an association between *MUC5B* promoter genotype and having ≥10 % of the lung occupied by HAAs (OR 1.1, 95 % CI 0.9–1.5, *P* = 0.33), or when we considered an alternate threshold of lung density (≥6.44 % in the FHS, OR 1.1, 95 % CI 0.7–1.6, *P* = 0.75). Finally, after adjusting for covariates, there was no evidence for an association between *MUC5B* promoter genotype and continuous measures of HAAs in the FHS (*P* = 0.31).

## Discussion

Our findings demonstrate that, although individuals with an increased percentage of the lung occupied by regions of high density are more likely to have interstitial lung abnormalities defined by visual assessment in both the COPDGene and the FHS cohorts, lung density thresholds do not appear to be strongly predictive of ILA defined by visual assessment. Although ILA defined by visual assessment and ILA defined by lung density thresholds both predict a phenotype that is associated with reduced total lung capacity, ILA defined by lung density thresholds are not associated with *MUC5B* promoter genotype.

As information accrues that suggests that the presence ILA on chest CT, in some cases, may define groups that are at an increased risk for the development of clinically evident pulmonary fibrosis [6], consistency in ILA detection across studies will be important to allow for accurate comparisons. Automated methods of lung abnormality detection are attractive in that they could provide a rapid, quantifiable, and reproducible method (at least across similar scanners and scanning protocols) [23] to determine if a person is at an increased risk for pulmonary fibrosis. Unfortunately, our findings suggest that most cases of ILA defined by visual assessment would be missed by detection methods based on classification using lung density alone. Given that the primary purpose of this research is to identify groups at an increased risk for pulmonary fibrosis, it is important to note that although both methods identify groups with reduced lung volumes (an effect noticeably greater with ILA detection methods based on lung density), the genetic factor most strongly associated with IPF (*MUC5B* promoter genotype) [15, 24–28] is similarly associated with ILA when defined by visual assessment [10] but not with ILA when defined by thresholds of lung density. This suggests that quantitative methods for detecting ILA based on lung density may include additional phenotypes associated with reduced total lung volume measurements (based on chest CT measurements) but not suggestive of an ILD (e.g. atelectasis, reduced inspiratory volume, or increased attenuation values due to increased soft tissue as can be noted in subjects with a high BMI).

As it has been known for some time that ILD is associated with increased measures of lung density [29], it is perhaps surprising that increased measures of lung density are not strongly predictive of ILA defined by visual assessment. To explore this phenomenon further, we postulated that additional factors known to influence lung density could modify the correlation between lung density metrics and ILA defined by visual assessment. We found that both chest CT-defined metrics of emphysema (a factor defined by reduced lung density) [19] and BMI (a factor that can increase image noise and thus lung density when increased) [30] influence the correlation between measures of lung density and ILA defined by visual assessment. In short, our findings are consistent with the fact that individuals with ILA defined by visual assessment will tend to have lower measures of high attenuation areas when a significant amount of emphysema is present. In addition, individuals with a high BMI, even without ILA defined by visual assessment, are more likely to have increased measures of high attenuation areas. Although measures of emphysema and BMI likely do not entirely explain the lack of a strong correlation between these methods of ILA detection, our findings do suggest that factors influencing global lung density can influence the performance of lung density thresholds when classifying pulmonary parenchymal abnormalities.

Our study has a number of important limitations. First, although our study suggests that there are limitations of lung density thresholds for the classification of ILA in multiple cohorts, longitudinal follow-up of individuals with ILA defined by various methods has not been presented. Second, while our study demonstrates that automated ILA classification methods (based on global increases in regions of high attenuating areas) alone may have limitations, our study does not exclude the possibility that alternate automated ILA detection methods (including those involving lung segmentation and texture analysis) [31] could have improved classification performance. Importantly, in patients with known interstitial lung disease, radiologic discrimination and quantitation methods based on texture analysis and feature selection have demonstrated good sensitivity and specificity when compared to radiologic interpretations [17, 18, 32, 33] and have been demonstrated to be superior to lung density based detection methods [33]. Third, although evidence has been presented that suggests, in some cases, that ILA defined by visual inspection could represent an early stage of pulmonary fibrosis, it is important to note that there is currently no “gold standard” criteria for the detection of an early stage of pulmonary fibrosis. In addition, some of the radiologic features that we have used to define ILA are atypical and suggest alternate diagnoses from idiopathic pulmonary fibrosis (e.g. centrilobular ground-glass nodules). Our findings do not preclude the possibility that alternate methods of visual assessment or quantitative measurement could ultimately, and more accurately, identify groups at high risk to develop pulmonary fibrosis. Fourth, it is worth noting that although specific HU thresholds for measurement of HAAs of the lung (between −600 and −250 HUs) exclude central pulmonary vasculature (~40 HU depending on hematocrit), it is not known what effect residual segmental and subsegmental vasculature (which might be captured by HAA thresholds) could have on HAA quantification. Finally, although our study raises concerns about the use of high attenuation lung density thresholds alone for the classification of ILA, our study does not exclude the possibility that measures of increased lung density could provide additive clinical information. In fact, our study demonstrates that increased measures of lung density are strongly correlated with reduced lung volumes, even in analyses limited to those with ILA defined by visual assessment. Future longitudinal studies will be needed to determine if increased measures of lung density among those with ILA are associated with a greater risk for a progressive disease.

## Conclusion

In conclusion, our findings demonstrate that metrics of increased lung density may be helpful in determining the severity of lung volume reduction, but alone, are insufficient in classifying interstitial lung abnormalities defined by visual assessment.

## Abbreviations

HAAs: high attenuation areas
ILA: interstitial lung abnormalities
ILD: interstitial lung disease
IPF: Idiopathic pulmonary fibrosis
CT: computed tomogram
FHS: Framingham Heart studies
UIP: usual interstitial pneumonia
DLCO: diffusion capacity of carbon monoxide
HUs: Hounsfield Units
TLC: total lung capacity
BMI: Body mass index
